# Rac1 and Stathmin but Not EB1 Are Required for Invasion of Breast Cancer Cells in Response to IGF-I

**DOI:** 10.1155/2011/615912

**Published:** 2011-09-25

**Authors:** Shigeru Morimura, Kazuhide Takahashi

**Affiliations:** Molecular Cell Biology Division, Kanagawa Cancer Center Research Institute, Yokohama 241-0815, Japan

## Abstract

Cell migration is considered necessary for the invasion that accompanies the directional formation of the cellular protrusions termed lamellipodia. In invasive breast cancer MDA-MB-231 cells, lamellipodia formation is preceded by translocation of the actin cytoskeletal regulatory protein WAVE2 to the leading edge. WAVE2 translocation and lamellipodia formation require many signaling molecules, including PI3K, Rac1, Pak1, IRSp53, stathmin, and EB1, but whether these molecules are necessary for invasion remains unclear. In noninvasive breast cancer MCF7 cells, no lamellipodia were induced by IGF-I, whereas in MDA-MB-231 cells, Rac1, stathmin, and EB1 were overexpressed. Depletion of Rac1 or stathmin by small interfering RNA abrogated the IGF-I-induced invasion of MDA-MB-231 cells; however, depletion of EB1 did not, indicating the necessity of Rac1 and stathmin but not EB1 for invasion. The signaling pathway leading to cell invasion may not be identical but shares some common molecules, leading to cell migration through lamellipodia formation.

## 1. Introduction

The formation of cellular protrusions such as lamellipodia at the leading edge of migrating cells is regulated by WASP/WAVE family of the actin cytoskeletal regulatory protein WAVE2 [1–3]. Before lamellipodia formation, WAVE2 is translocated to the leading edge along microtubules [4–6], which is mediated by many signaling and regulatory molecules. WAVE2 forms a complex with IQGAP1, the motor protein kinesin1 [6, 7], Pak1 [8], and IRSp53 [9] in the cytoplasm of quiescent cells and gathers additional IQGAP1 and kinesin1 [6], which are released from the Rac1-CLIP-170 complex [7], after stimulation of cells with HGF or IGF-I. Concomitantly, WAVE2-bound Pak1 is Rac1-dependently activated, which in turn inactivates stathmin, a microtubule-destabilizing protein [10, 11], by phosphorylation [8]. Stathmin is constitutively associated with the microtubule-end-binding protein EB1 [12], and the phosphorylated stathmin-EB1 complex is recruited to the microtubule ends that bear the WAVE2 complex after IGF-I stimulation [8]. Following translocation to the leading edge, WAVE2 is captured by PtdInsP_3 _through WAVE2-bound IRSp53 [13]. PtdInsP_3_ is produced by PI3K near the IGF-I receptor IGF-IR that is locally activated in the membrane region facing IGF-I [13]. These results indicate that many signaling and regulatory molecules, including IGF-IR, PI3K, Rac1, Pak1, IRSp53, stathmin, and EB1, are involved in inducing the directional lamellipodia formation in migrating cells. However, whether these molecules, except for WAVE2 [14], are crucial for invasion of MDA-MB-231 cells remains unclear. 

 We report here that Rac1, stathmin, and EB1 were overexpressed in invasive breast cancer MDA-MB-231 cells compared to noninvasive breast cancer MCF7 cells. In MCF7 cells, no lamellipodia formation was induced, and Rac1 was not activated by IGF-I. Expression and activation of other molecules were not significantly different between the cell lines. Depletion of Rac1 and stathmin but not EB1 by RNA interference resulted in significant inhibition of the IGF-I-induced invasion of MDA-MB-231 cells. These results indicate that Rac1, stathmin, and EB1 are overexpressed in invasive MDA-MB-231 cells, whereas Rac1 and stathmin but not EB1 are required for invasion of the cells in response to IGF-I.

## 2. Materials and Methods

### 2.1. Cell Culture

Human breast cancer MDA-MB-231 and MCF7 cells were obtained from American Type Culture Collection (Manassas, VA) and maintained as described earlier [9, 13]. Before stimulation with 50 ng/mL IGF-I (Peprotech, London, UK), cells were serum-starved by incubation in medium containing 0.1% FBS for 16 h.

### 2.2. Lamellipodia Formation and WAVE2 Translocation Assays

Cells grown on glass slide chambers (BD Falcon, Bedford, MA) were stained with rhodamine-phalloidin (Invitrogen, Carlsbad, CA) or anti-WAVE2 antibody (Santa Cruz Biotechnology, Santa Cruz, CA). For quantification of lamellipodia formation and WAVE2 translocation, the frequency of cells with lamellipodia or WAVE2 staining at the leading edge of cells was counted as described earlier [6, 8, 9, 13].

### 2.3. Cell Invasion Assay

Cell invasion assay was performed, using an invasion chamber (24-well, 8-*μ*m pore size; BD Biosciences, Bedford, MA). Cells in medium containing 0.1% FBS (low-serum medium) were placed on the chamber insert membrane coated with the basement membrane matrix and incubated for 6 h toward the low-serum medium containing or lacking 50 ng/mL IGF-I. Cells that invaded through the pores and spread over the bottom surface of the chamber membrane were stained with Giemsa solution, and the number of invaded cells in 3 × 3 mm area on the membrane was determined [7].

### 2.4. Immunoprecipitation and Immunoblot Analysis

Cells were lysed in RIPA buffer with a cell disruptor [6, 8], and proteins in the cell lysates were immunoprecipitated with antibodies to the p85 subunit of PI3K, Rac1 (Millipore, Temecula, CA), and active Rac1 (NewEast Biosciences, Malvern, PA), followed by the slurry of Protein A-Sepharose (GE Healthcare, Uppsala, Sweden). The SDS-PAGE and immunoblotting procedures were performed as described in detail earlier [8, 9, 13] using antibodies to IGF-IR, Pak1, stathmin, EB1, IRSp53 (Santa Cruz Biotechnology), PI3K p85, Rac1, and WAVE2 (Millipore).

### 2.5. Immunofluorescence Analysis

Cells on glass slides were fixed, permeabilized, blocked, and stained with rhodamine-conjugated phalloidin or antibodies to phospho-IGF-IR (Tyr1135/1136) (Sigma-Aldrich, St. Louis, MO) and WAVE2 [13].

### 2.6. PI3K Activity Assay

PI3K activity was measured by immunoprecipitation of PI3K p85 with anti-p85 antibody, followed by the kinase reaction of the immunoprecipitates in a PI3K activity ELISA kit (Echelon Bioscience, Salt Lake City, UT), according to the manufacturer's instructions.

### 2.7. Rac1 Activity Assay

Rac1 activity was measured by immunoprecipitation with antibody to active-form GTP-bound Rac1 (NewEast Biosciences). Immunoprecipitation of active Rac1 was performed for 4- to 8-fold concentrated cell lysates compared to total Rac1. The immunoprecipitates were subjected to SDS-PAGE and immunoblotted with anti-Rac1 antibody.

### 2.8. RNA Interference Assay

Cells were incubated with 50 nM of mismatch negative control small interfering RNA (Dharmacon) or 2 different small interfering RNAs of Rac1, stathmin, or EB1 (Invitrogen) for 48 h using RNAiMAX (Invitrogen). The target sequences of human Rac1 (RAC1), stathmin (STMN1) [8], and EB1 (MAPRE1) [13] used were 5′-CACCTCAGGATACCACTTTGCACGG-3′ (Rac1-1), 5′-TATCCCATA-AGCCCAGATTCACCGG-3′ (Rac1-2), 5′-TTGACCGAGGGCTGAGAATCAGCTC-3′ (stathmin-1), 5′-CTTTCACCTGGATATCAGAAGAAGC-3′ (stathmin-2), 5′-TGCT-AGAAGTGAGAGGTTTCTTCGG-3′ (EB1-1), and 5′-TTCAACTGCAGAGACTCA-TTGATCC-3′ (EB-1-2), respectively. Cell viability was determined using a LIVE/DEAD Fixable Dead Cell Stain kit (Invitrogen). To assess the efficiency of small interfering RNA, the same amounts of total cell lysates in SDS-PAGE buffer were resolved by SDS-PAGE for immunoblot analysis with antibodies to *β*-actin (Sigma-Aldrich) and Rac1, stathmin, or EB1.

### 2.9. Statistical Analysis

Statistical significance between the mean values of triplicate assays was calculated using the unpaired Student's *t*-test. Data were considered significant at a *P* value of less than 0.05.

## 3. Results

### 3.1. Distinct Phenotypes of Invasion and Lamellipodia Formation between MDA-MB-231 and MCF7 Cells in Response to IGF-I

**Figure 1:**
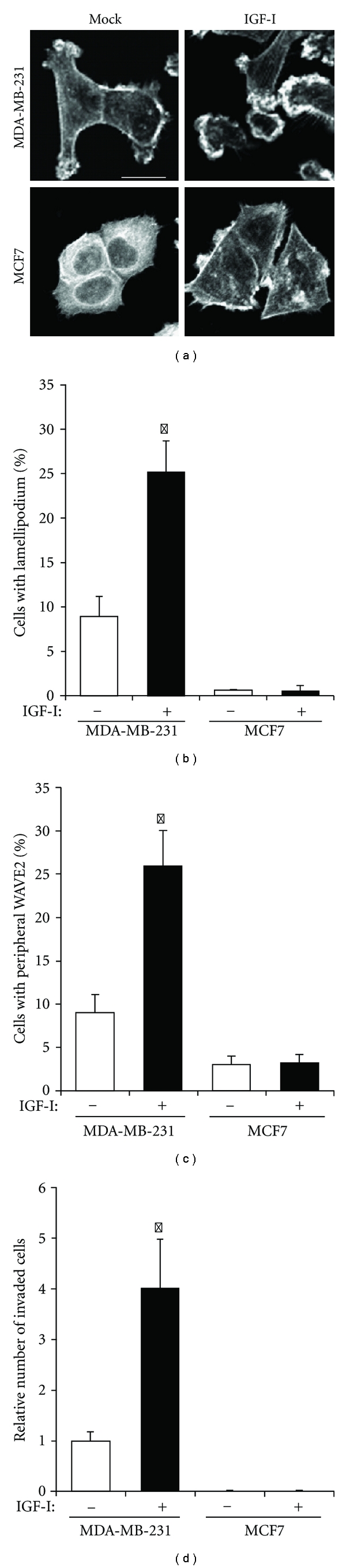
Induction of lamellipodia formation and invasion by IGF-I in MDA-MB-231 cells but not MCF7 cells. (a) After stimulation with (IGF-I) or without (mock) IGF-I, cells were stained with phalloidin. Scale Bar 20 *μ*m. (b) Cells after stimulation with (+) or without (−) IGF-I were stained with phalloidin, and the frequency of cells with lamellipodia was determined. The mean (SD) values of triplicate experiments are given. *P < 0.003, by Student's *t*-test. (c) After stimulation with (+) or without (−) IGF-I, cells were stained with anti-WAVE2 antibody, and the frequency of cells with peripheral WAVE2 staining was determined. The means (SD) of triplicate experiments are given. *P < 0.0001. (d) After incubation of cells toward medium containing (+) or lacking (−) IGF-I, invaded cells were stained with Giemsa solution. The number of invaded cells relative to quiescent MDA-MB-231 cells was determined, and the mean (SD) values of triplicate experiments are given. *P < 0.004.

To determine the cellular abilities of lamellipodia formation and invasion in response to IGF-I, we conducted assays for lamellipodia formation, WAVE2 translocation, and cell invasion for MDA-MB-231 and MCF7 cells. Phalloidin staining of cells revealed that the F-actin framework in quiescent MDA-MB-231 cells was rearranged to form lamellipodia at the leading edge after IGF-I stimulation (Figure 1(a)). In contrast, a fine F-actin meshwork over the cytoplasm of quiescent MCF7 cells formed a distinct F-actin framework or local aggregates in the cell periphery after IGF-I stimulation (Figure 1(a)). Although the frequency of lamellipodia in MDA-MB-231 cells was significantly increased by IGF-I (*P* < 0.003), it remained significantly low in MCF7 cells after IGF-I stimulation (Figure 1(b)). Similarly, WAVE2 translocation to the leading edge was significantly promoted by IGF-I in MDA-MB-231 cells (*P* < 0.0001; Figure 1(c)). However, the frequency of cells with peripheral WAVE2 was very low in both quiescent and IGF-I-stimulated MCF7 cells (Figure 1(c)). When cells were incubated on invasion chamber membranes coated with the basement membrane matrix, the mean number of invaded MDA-MB-231 cells after incubation for 6 h toward IGF-I-containing medium was 1,382 in 3 × 3 mm area and significantly larger than 347 in quiescent cells incubated toward the low-serum medium lacking IGF-I (*P* < 0.004; Figure 1(d)). In contrast, practically no invaded MCF7 cells were observed either before (12) or after IGF-I stimulation (10) (Figure 1(d)).

### 3.2. Comparable Expression and Activation of IGF-IR and PI3K in MDA-MB-231 and MCF7 Cells

**Figure 2:**
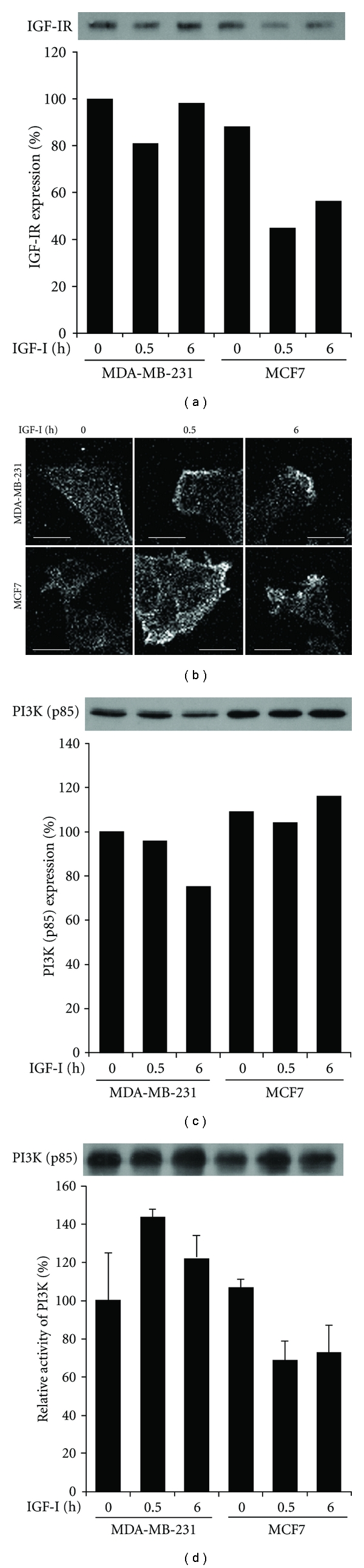
Expression and activation of IGF-IR and PI3K in MDA-MB-231 and MCF7 cells. (a) After stimulation with IGF-I for 0, 0.5, or 6 h, cells were lysed and processed for immunoblotting with anti-IGF-IR antibody. Values are given as band intensity relative to that in unstimulated control MDA-MB-231 cells. (b) After stimulation with IGF-I, cells were immunostained with antibody to phospho-IGF-IR (Tyr1135/1135). Scale Bars 20 *μ*m. (c) After stimulation with IGF-I, cells were lysed and processed for immunoblotting with anti-PI3K (p85) antibody. Values are given as band intensity relative to that in unstimulated control MDA-MB-231 cells. (d) Cells after stimulation with IGF-I were lysed and immunoprecipitated with anti-PI3K (p85) antibody. PI3K activity was assayed for the immunoprecipitates as described in Materials and Methods. The mean (SD) values of triplicate assays are given as activity relative to that in unstimulated control MDA-MB-231 cells.

To clarify the differences in the signaling pathways that lead to WAVE2 translocation and lamellipodia formation in the 2 breast cancer cell lines, we first examined the expression and activation of IGF-IR at 0.5 and 6 h after IGF-I stimulation. The expression level of IGF-IR was nearly constant in MDA-MB-231 cells before and after IGF-I stimulation, whereas in MCF7 cells, it decreased after IGF-I stimulation to around 50% of that in quiescent MDA-MB-231 cells (Figure 2(a)). We carried out immunofluorescence assay using the antibody to phospho-IGF-IR at Tyr1135/1136 (p-IGF-IR) to assess IGF-IR activation [15]. The results revealed that p-IGF-IR was locally distributed at the leading edge of MDA-MB-231 cells after IGF-I stimulation for 0.5 or 6 h (Figure 2(b)). In MCF7 cells, p-IGF-IR was distributed at the tips of short cell protrusions after IGF-I stimulation (Figure 2(b)). These results indicate the local activation of IGF-IR in both cell lines in response to IGF-I.

 Because PI3K is recruited to and activated by the activated IGF-IR, we next compared the expression and activation of PI3K in the 2 cell lines. Immunoblot analysis revealed that expression levels of the p85 subunit of PI3K were comparable in the cell lines before and after IGF-I stimulation (Figure 2(c)). PI3K activity was comparable between the 2 quiescent cell lines, whereas activity increased by 20–40% in MDA-MB-231 cells and decreased by 30% in MCF7 cells during incubation with IGF-I (Figure 2(d)).

### 3.3. IGF-I Activates Rac1 That Is Overexpressed in MDA-MB-231 Cells

**Figure 3:**
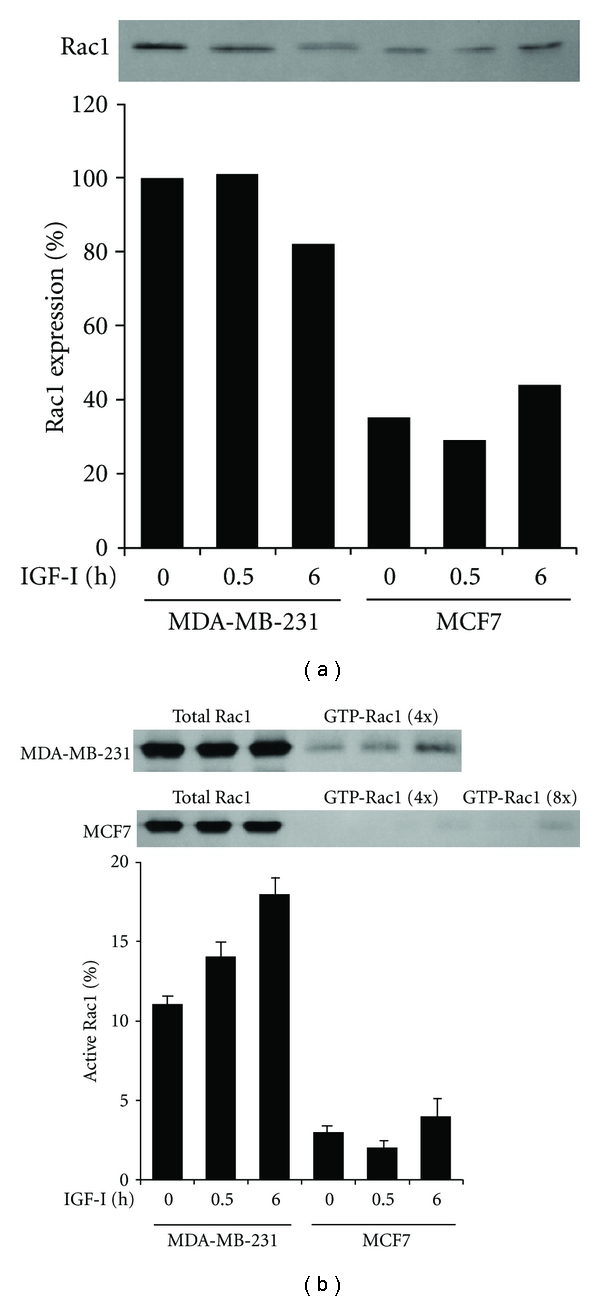
Overexpression and activation of Rac1 by IGF-I in MDA-MB-231 but not in MCF7 cells. (a) Cells after stimulation with IGF-I for 0, 0.5, or 6 h were lysed and processed for immunoblotting with anti-Rac1 antibody. Band intensity was measured and values are given as Rac1 expression relative to that in unstimulated control MDA-MB-231 cells. (b) After stimulation with IGF-I, total and activated Rac1 were immunoprecipitated from the cell lysates and 4- or 8-fold concentrated cell lysates, respectively, and processed for immunoblotting with anti-Rac1 antibody. Band intensity was measured and the mean (SD) values of triplicate experiments are given as the amount of active Rac1 relative to total Rac1.

The GTPase Rac1 is activated by PI3K through PtdInsP_3_ binding to RacGEF [16]. To determine whether the PI3K activation signal is transmitted to Rac1, we compared Rac1 expression and activation in the 2 cell lines. Whereas Rac1 expression in MDA-MB-231 cells decreased slightly after IGF-1 stimulation for 6 h, it remained low in MCF7 cells before and after IGF-I stimulation, at a range of 29–43% of that in quiescent MDA-MB-231 cells (Figure 3(a)). The relative amount of active Rac1 to total Rac1 in MDA-MB-231 cells gradually increased from 11% to 14% and 18% after IGF-I stimulation for 0.5 and 6 h, respectively (Figure 3(b)). In contrast, only a trace amount of Rac1 was activated by IGF-I in MCF7 cells (Figure 3(b)).

### 3.4. Comparable Expression of Pak1, IRSp53, and WAVE2 in MDA-MB-231 and MCF7 Cells

**Figure 4:**
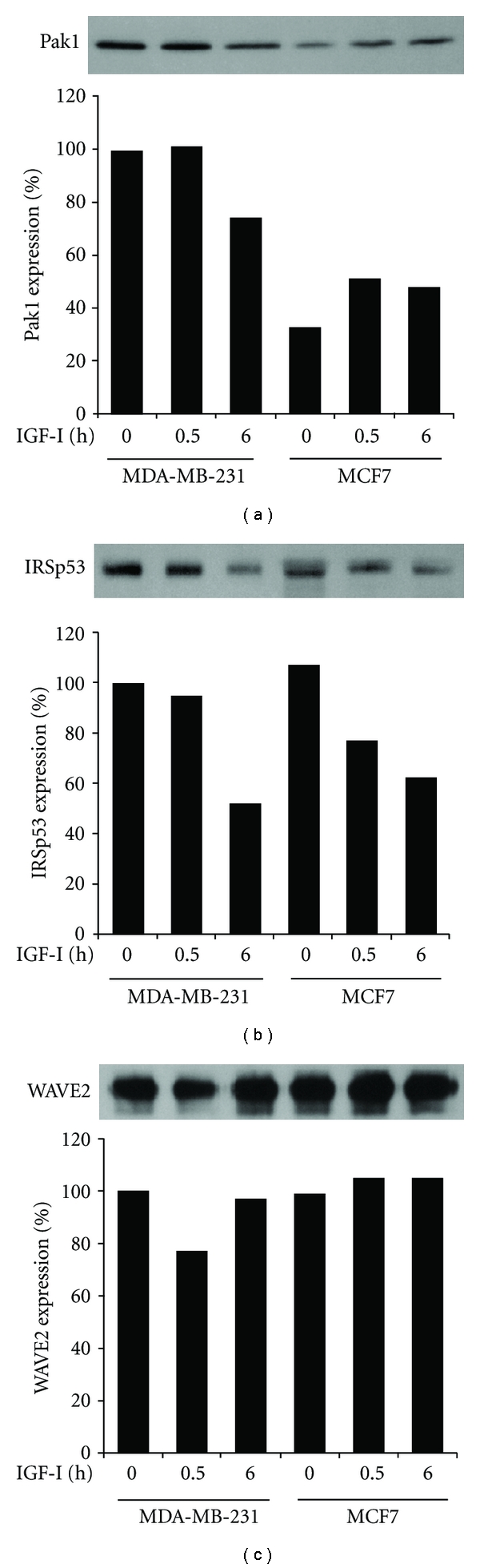
Expression of Pak1, IRSp53, and WAVE2 in MDA-MB-231 and MCF7 cells. After stimulation with IGF-I for 0, 0.5, or 6 h, cells were lysed and processed for immunoblotting with antibodies to Pak1 (a), IRSp53 (b), and WAVE2 (c). Band intensity relative to that in unstimulated control MDA-MB-231 cells is given.

Pak1 is a downstream effector of Rac1 [17] and is constitutively associated with WAVE2 in MDA-MB-231 cells [8]. Whereas the expression level of Pak1 decreased slightly in MDA-MB-231 cells after a 6 h incubation with IGF-I, it remained low in MCF7 cells before and after IGF-I stimulation, at a range of 33–51% of that in quiescent MDA-MB-231 cells (Figure 4(a)). After translocation to the leading edge, WAVE2 is linked to PtdInsP_3_ by WAVE2-bound IRSp53 [9, 13]. Immunoblot analysis revealed that IRSp53 expression was similarly reduced in both cell lines during IGF-I stimulation, retaining comparable levels (Figure 4(b)). WAVE2 expression levels in the 2 cell lines were comparable and remained nearly constant throughout the incubation with or without IGF-I (Figure 4(c)).

### 3.5. Overexpression of Stathmin and EB1 in MDA-MB-231 Cells Compared to MCF7 Cells

**Figure 5:**
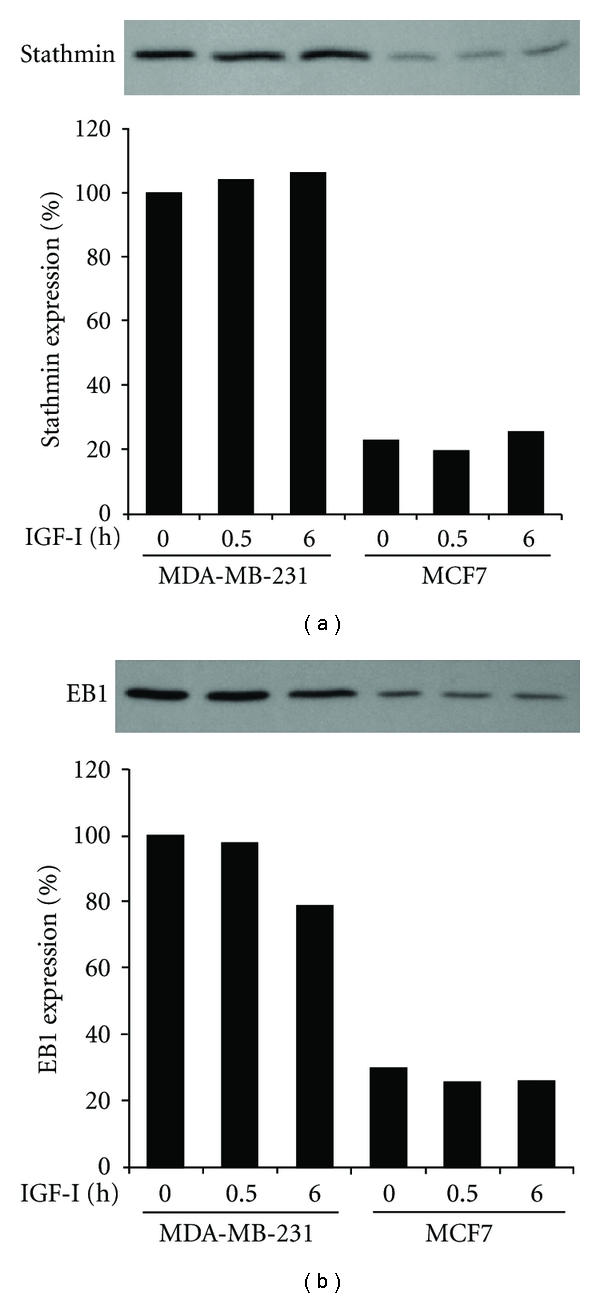
Overexpression of stathmin and EB1 in MDA-MB-231 cells compared to MCF7 cells. Cells after stimulation with IGF-I for 0, 0.5, or 6 h were lysed and processed for immunoblotting with antibodies to stathmin (a) and EB1 (b). Band intensity was measured, and values are given as expression relative to that in unstimulated control MDA-MB-231 cells.

Upon stimulation of MDA-MB-231 cells with IGF-I, Pak1 is activated and in turn phosphorylates stathmin, thereby leading to recruitment of the phosphorylated stathmin-EB1 complex to the microtubule ends, which bear the WAVE2 complex [13]. Expression levels of stathmin remained nearly constant in both cell lines before and after IGF-I stimulation; however, they were low in MCF7 cells (22–26%) of that in quiescent MDA-MB-231 cells (Figure 5(a)). Similar to stathmin, EB1 expression levels in MCF7 cells before and after IGF-I stimulation were less than 30% of those in quiescent MDA-MB-231 cells (Figure 5(b)).

### 3.6. Rac1 and Stathmin but Not EB1 Are Required for Invasion of MDA-MB-231 Cells in Response to IGF-I

**Figure 6:**
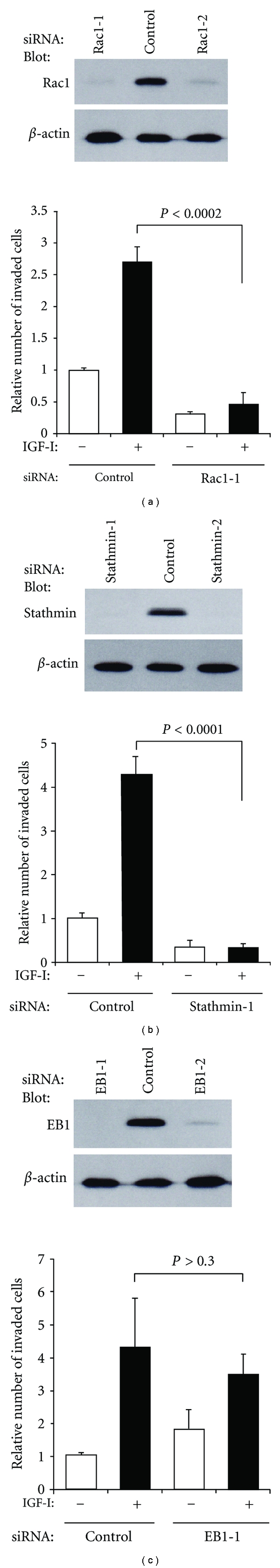
Rac1 and stathmin but not EB1 are required for IGF-I-induced invasion of MDA-MB-231 cells. Cells after incubation for 48 h with mismatch negative control small interfering RNA and 2 different small interfering RNAs of Rac1 (a), stathmin (b), or EB1 (c) were lysed and the same amounts of total cell lysates were immunoblotted with antibodies to *β*-actin and Rac1 (a), stathmin (b), or EB1 (c). Cells transfected with control small interfering RNA and Rac1-1 (a), stathmin-1 (b), or EB1-1 small interfering RNA (c) were incubated for 6 h toward medium containing (+) or lacking (−) IGF-I. The mean (SD) values of triplicate assays are given as the number of invaded cells relative to that in unstimulated control MDA-MB-231 cells.

Rac1 was activated by IGF-I in MDA-MB-231 cells but not in MCF7 cells (Figure 3(b)), and expression levels of Rac1, stathmin, and EB1 were significantly higher in MDA-MB-231 cells than those in MCF7 cells (Figures 3(a), 5(a), and 5(b)). To explore the necessity of these proteins for IGF-I-induced invasion of MDA-MB-231 cells, we studied suppression of each of these proteins by 2 different small interfering RNAs (Figure 6). When Rac1 expression was depleted by Rac1-1 small interfering RNA, the mean number of invaded cells was very small before (104) and after IGF-I stimulation (157), contrary to control cultures in which it increased from 335 to 906 by IGF-I. In IGF-I-stimulated cells, the relative number of invaded Rac1-deficient cells was significantly smaller than that of control cells (*P* < 0.0002) (Figure 6(a)). Similarly, the mean number of invaded cells was very small both before (109) and after IGF-I stimulation (109) in stathmin-deficient cells that were transfected with stathmin-1 small interfering RNA. By contrast, the mean number of invaded cells transfected with control small interfering RNA significantly increased from 315 to 1,356 by IGF-I stimulation. Consequently, the relative number of invaded cells after IGF-I stimulation was significantly smaller in stathmin-deficient cells than that in control cells (*P* < 0.0001; Figure 6(b)). Contrary to these, the mean number of invaded cells in EB1-deficient quiescent culture was significantly larger (574) than that in control quiescent culture (312, *P* < 0.04). In addition, IGF-I stimulation caused a significant increase in the mean number of invaded cells in both control (1,341, *P* < 0.005) and EB1-deficient cultures (1,082, *P* < 0.02). Therefore, the values were not significantly different from each other in IGF-I-stimulated cultures (*P* > 0.3; Figure 6(c)).

## 4. Discussion

Assays for lamellipodia formation, WAVE2 translocation, and cell invasion revealed that IGF-I induced significant increases in the frequencies of lamellipodia formation, WAVE2 translocation, and cell invasion in MDA-MB-231 cells. In contrast, IGF-I failed to induce lamellipodia formation, WAVE2 translocation, or invasion in MCF7 cells. This suggests that MDA-MB-231 cells have the ability to invade through the invasion chamber membranes and that MCF7 cells do not form lamellipodia nor invade in response to IGF-I.

 The signaling pathway leading to lamellipodia formation through WAVE2 translocation in MDA-MB-231 cells is mediated by many molecules that include IGF-IR, PI3K, Rac1, Pak1, IRSp53, stathmin, and EB1 [8, 9, 13]. All of these molecules are essential for IGF-I-induced lamellipodia formation, but whether they are also necessary for invasion was unclear, except for WAVE2 [14]. Comparison of MDA-MB-231 and MCF7 cells revealed that the expression and activation of IGF-IR and PI3K are not significantly different in the 2 cell lines before and after IGF-I stimulation. This suggests that both cell lines respond to IGF-I and produce PtdInsP_3_, dependent on PI3K. The small GTPase Rac1, a key inducer of lamellipodia [18] and a downstream effector of PI3K [16], was overexpressed and activated by IGF-I in MDA-MB-231 cells but not MCF7 cells, in the latter of which Rac1 was hardly activated by IGF-I. A possible explanation for the lack of Rac1 activation in MCF7 cells is reduced expression of RacGEF, but the actual reason for the lack of Rac1 activation remains unknown in the current study. A crucial role of Rac1 in the IGF-I-induced invasion of MDA-MB-231 cells was clearly demonstrated by RNA interference assay. Rac1 depletion significantly suppressed the frequency of invasion in both quiescent and IGF-I-stimulated MDA-MB-231 cells. This indicates the necessity of Rac1 for IGF-I-induced cell invasion in the cells. Overexpression of Rac1 has been reported in breast carcinomas [19] and may be involved in the invasion of gliomas [20] and macrophages [21].

 The WAVE2 complex, including Pak1 [8] and IRSp53 [9], is translocated along microtubules following stathmin phosphorylation and EB1-mediated binding of the phosphorylated stathmin-EB1 complex to the microtubule ends that bear the WAVE2 complex [8, 13]. Both stathmin inactivation by phosphorylation [22] and EB1 binding to the microtubule ends are considered to promote the persistent microtubule growth [23]. However, depletion of stathmin or EB1 in MDA-MB-231 cells differentially affected the invasive potential of the cells. Stathmin depletion significantly inhibited the IGF-I-induced invasion of MDA-MB-231 cells, whereas EB1 depletion did not. This suggests that stathmin but not EB1 is essential for MDA-MB-231 cell invasion in response to IGF-I. Because binding of the stathmin-EB1 complex to the microtubule ends is mediated by EB1 [13], not only EB1 but also the binding of stathmin to the microtubule ends may be unnecessary for MDA-MB-231 cell invasion. Overexpression of stathmin has been reported in breast carcinomas, sarcomas, and hepatomas [24–26] and may be involved in tumor invasion or metastatic potential [25, 26]. 

 In conclusion, Rac1, stathmin, and EB1 are overexpressed in MDA-MB-231 cells compared to MCF7 cells, which do not form lamellipodia nor invade in response to IGF-I. Among these proteins, Rac1 and stathmin but not EB1 are also required for IGF-I-induced invasion of MDA-MB-231 cells. These results suggest that the signaling pathway leading to cell invasion is not identical but shares some common molecules, leading to cell migration through lamellipodia formation. Further investigation of the signaling molecules that are required and sufficient for cell invasion may increase understanding of the regulation of cell invasion and metastasis.

## References

[1] Miki H, Suetsugu S, Takenawa T (1998). WAVE, a novel WASP-family protein involved in actin reorganization induced by Rac. *EMBO Journal*.

[2] Machesky LM, Mullins RD, Higgs HN (1999). Scar, a WASp-related protein, activates nucleation of actin filaments by the Arp2/3 complex. *Proceedings of the National Academy of Sciences of the United States of America*.

[3] Svitkina TM, Borisy GG (1999). Arp2/3 complex and actin depolymerizing factor/cofilin in dendritic organization and treadmilling of actin filament array in lamellipodia. *Journal of Cell Biology*.

[4] Oikawa T, Yamaguchi H, Itoh T (2004). Ptdlns(3,4,5)P3 binding is necessary for WAVE2-induced formation of lamellipodia. *Nature Cell Biology*.

[5] Leng Y, Zhang J, Badour K (2005). Abelson-interactor-1 promotes WAVE2 membrane translocation and Abelson-mediated tyrosine phosphory lation required for WAVE2 activation. *Proceedings of the National Academy of Sciences of the United States of America*.

[6] Takahashi K, Suzuki K (2008). Requirement of kinesin-mediated membrane transport of WAVE2 along microtubules for lamellipodia formation promoted by hepatocyte growth factor. *Experimental Cell Research*.

[7] Suzuki K, Takahashi K (2008). Regulation of lamellipodia formation and cell invasion by CLIP-170 in invasive human breast cancer cells. *Biochemical and Biophysical Research Communications*.

[8] Takahashi K, Suzuki K (2009). Membrane transport of WAVE2 and lamellipodia formation require Pak1 that mediates phosphorylation and recruitment of stathmin/Op18 to Pak1-WAVE2-kinesin complex. *Cellular Signalling*.

[9] Takahashi K, Suzuki K (2010). WAVE2 targeting to phosphatidylinositol 3,4,5-triphosphate mediated by insulin receptor substrate p53 through a complex with WAVE2. *Cellular Signalling*.

[10] Belmont LD, Mitchison TJ (1996). Identification of a protein that interacts with tubulin dimers and increases the catastrophe rate of microtubules. *Cell*.

[11] Cassimeris L (2002). The oncoprotein 18/stathmin family of microtubule destabilizers. *Current Opinion in Cell Biology*.

[12] Mimori-Kiyosue Y, Tsukita S (2003). “Search-and-capture” of microtubules through plus-end-binding proteins (+TIPs). *Journal of Biochemistry*.

[13] Takahashi K, Tanaka T, Suzuki K (2010). Directional control of WAVE2 membrane targeting by EB1 and phosphatidylinositol 3,4,5-triphosphate. *Cellular Signalling*.

[14] Kurisu S, Suetsugu S, Yamazaki D, Yamaguchi H, Takenawa T (2005). Rac-WAVE2 signaling is involved in the invasive and metastatic phenotypes of murine melanoma cells. *Oncogene*.

[15] Motley ED, Kabir SM, Gardner CD (2002). Lysophosphatidylcholine inhibits insulin-induced Akt activation through protein kinase C-*α* in vascular smooth muscle cells. *Hypertension*.

[16] Fleming IN, Gray A, Downes CP (2000). Regulation of the Rac1-specific exchange factor Tiam1 involves both phosphoinositide 3-kinase-dependent and -independent components. *Biochemical Journal*.

[17] Manser E, Leung T, Monfries C, Teo M, Hall C, Lim L (1992). Diversity and versatility of GTPase activating proteins for the p21rho subfamily of ras G proteins detected by a novel overlay assay. *Journal of Biological Chemistry*.

[18] Hall A (1998). Rho GTpases and the actin cytoskeleton. *Science*.

[19] Schnelzer A, Prechtel D, Knaus U (2000). Rac1 in human breast cancer: overexpression, mutation analysis, and characterization of a new isoform, Rac1b. *Oncogene*.

[20] Chan AY, Coniglio SJ, Chuang YY (2005). Roles of the Rac1 and Rac3 GTPases in human tumor cell invasion. *Oncogene*.

[21] Wheeler AP, Wells CM, Smith SD (2006). Rac1 and Rac2 regulate macrophage morphology but are not essential for migration. *Journal of Cell Science*.

[22] Wittmann T, Bokoch GM, Waterman-Storer CM (2004). Regulation of microtubule destabilizing activity of Op18/stathmin downstream of Rac1. *Journal of Biological Chemistry*.

[23] Komarova Y, De Groot CO, Grigoriev I (2009). Mammalian end binding proteins control persistent microtubule growth. *Journal of Cell Biology*.

[24] Curmi PA, Noguès C, Lachkar S (2000). Overexpression of stathmin in breast carcinomas points out to highly proliferative tumours. *British Journal of Cancer*.

[25] Hsieh SY, Huang SF, Yu MC (2010). Stathmin1 overexpression associated with polyploidy, tumor-cell invasion, early recurrence, and poor prognosis in human hepatoma. *Molecular Carcinogenesis*.

[26] Belletti B, Nicoloso MS, Schiappacassi M (2008). Stathmin activity influences sarcoma cell shape, motility, and metastatic potential. *Molecular Biology of the Cell*.

